# Real-World Three-Year Clinical Outcomes of Biolimus-Eluting Stents versus Other Contemporary Drug-Eluting Stents in Patients with Acute Myocardial Infarction Patients: Data from the Korea Acute Myocardial Infarction Registry (KAMIR)

**DOI:** 10.1155/2021/6698582

**Published:** 2021-07-20

**Authors:** Ji Young Park, Seung-Woon Rha, Yung-Kyun Noh, Byoung Geol Choi, Ji Yeon Hong, Jae-Woong Choi, Sung Kee Ryu, Sung-Hun Park, Yong Hoon Kim, Myung Ho Jeong

**Affiliations:** ^1^Division of Cardiology, Department of Internal Medicine, Nowon Eulji Medical Center, Eulji University, School of Medicine, Seoul, Republic of Korea; ^2^Division of Cardiology, Department of Internal Medicine, Korea University Guro Hospital, Seoul, Republic of Korea; ^3^Department of Computer Science, Hanyang University, Seoul, Republic of Korea; ^4^School of Computational Sciences, Korea Institute for Advanced Study, Seoul, Republic of Korea; ^5^Division of Cardiology, Department of Internal Medicine, Kangwon National University School of Medicine, Chuncheon, Republic of Korea; ^6^Division of Cardiology, Department of Internal Medicine, Chonnam National University Hospital, Gwangju, Republic of Korea

## Abstract

**Introduction:**

Biolimus-eluting stents (BES) are known to be superior to bare-metal stents. This study aims to evaluate the safety and efficacy of BES compared to other drug-eluting stents (DES) based on big data from the Korea Acute Myocardial Infarction Registry (KAMIR).

**Methods:**

The study analyzed a total of 9,759 acute myocardial infarction (AMI) patients who underwent percutaneous coronary intervention (PCI) with DES. Total death, cardiac death, recurrent MI, revascularization, stent thrombosis, target lesion failure (TLF, composite of cardiac death, recurrent myocardial infarction (MI), and target lesion revascularization), and major adverse cardiac events (MACE, composite of total death, recurrent MI, and revascularization) were analyzed in patients with AMI up to three years. Study populations were divided into BES (*n* = 2,020), everolimus-eluting stents (EES, *n* = 5,293), and zotarolimus-eluting stents (ZES, *n* = 2,446) groups.

**Results:**

To adjust baseline potential confounders, an inverse probability weighting (IPTW) analysis was performed. After IPTW, at three years, total death (7.2%, 8.6%, and 9.5%, *P* < 0.001), cardiac death (4.1%, 5.3%, and 6.6%, *P* < 0.001), recurrent MI (1.6%, 2.6%, and 3.2%, *P* < 0.001), TLF (6.5%, 8.1%, and 9.1%, *P* < 0.001), and MACE (15.8%, 17.5%, and 18.2%, *P* < 0.001) were lowest in the BES group compared with the other DES groups in AMI patients. During the 3-year clinical follow-up, the BES group showed better outcomes of MACE (hazard ratio (HR), 0.773; 95% confidence interval (CI), 0.676–0.884; *P* < 0.001), TLF (HR, 0.659; 95% CI, 0.538–0.808; *P* < 0.001), total death (HR, 0.687; 95% CI, 0.566–0.835; *P* < 0.001), and cardiac death (HR,0.593; 95% CI, 0.462–0.541; *P* < 0.001) than the EES groups.

**Conclusions:**

In this study, BES was superior to EES or ZES in reducing total death, cardiac death, TLF, and MACE in AMI patients.

## 1. Introduction

Durable polymers of first-generation drug-eluting stents (DESs) have safety issues, such as very late stent thrombosis (ST), which is related to adverse clinical outcomes [1]. Recently, biodegradable polymer-coated DESs were reported to be better in reducing very late ST and improving clinical outcomes than first-generation DES [2]. Biolimus-eluting stents (BES) are polymer-free and carrier-free drug-coated stents that transfer umirolimus (also known as biolimus A9), a highly lipophilic sirolimus analogue, into the vessel wall over a one-month period [3]. In the Prospective Randomized Comparison of the BioFreedom Biolimus A9 Drug-Coated Stent versus the Gazelle Bare-Metal Stent in Patients at High Bleeding Risk (LEADERS FREE) trial, the use of BES in patients with a high risk of bleeding who underwent percutaneous coronary intervention (PCI) reduces the incidence of target lesion revascularization (TLR) and is superior to bare-metal stents in terms of safety and efficacy [4].

In this study, we compared the three-year clinical outcomes between BES and other DESs, including everolimus-eluting stents (EES) and zotarolimus-eluting stents (ZES) in patients with acute myocardial infarction (AMI). The study was based on big data from the Korea AMI registry (KAMIR).

## 2. Materials and Methods

### 2.1. Study Population

**Figure 1:**
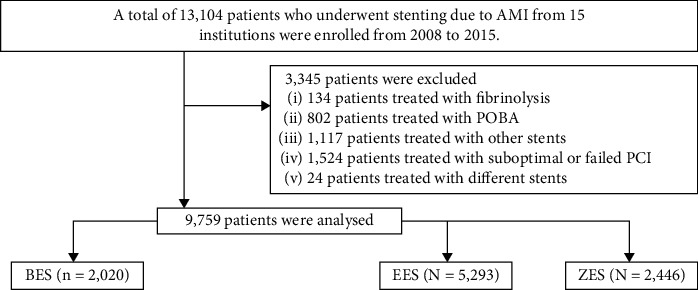
Study population. PCI, percutaneous coronary intervention; POBA, plain old balloon angioplasty; DESs, drug-eluting stents; BES, biolimus-eluting stent; EES, everolimus-eluting stents; ZES, zotarolimus-eluting stents.

The study population is described in Figure 1. A total of 13,104 patients who underwent PCI at 15 different institutions were enrolled from 2008 to 2015. Patients treated with fibrinolysis (*n* = 134), plain old balloon angioplasty (POBA, *n* = 802), other DESs (*n* = 1,117), suboptimal or failed PCI (*n* = 1,524), and different DES (*n* = 24) were excluded.

A total of 9,759 patients were analyzed, and 2,020 patients were treated with BES. Of those, 2,020 patients were treated with BES. Of those, 1,488 patients were treated with Biomatrix (Biosensors international, Morges, Switzerland) and 532 patients were treated with Nobori (Terumo Corporation, Tokyo, Japan). 5,293 patients were treated with EES (Xience Prime stent, Abbott Vascular, Santa Clara, CA; Promus Element stent, Boston Scientific, Natick, MA) and 2,446 patients were treated with ZES (Resolute Integrity stent; Medtronic, Inc., Minneapolis, MN). Figure 1 shows the three main groups of BES (*n* = 2,020), EES (*n* = 5,293), and ZES (*n* = 2,446). In this study, data have been collected after obtaining written informed consent prior to enrolment and a three-year clinical follow-up was completed by face-to-face interviews, phone calls, or chart review.

### 2.2. Clinical Outcomes and Study Definitions

In this study, we compared the three-year clinical outcomes, such as total death, cardiac death, recurrent MI, coronary revascularization, ST, target lesion failure (TLF), and major adverse cardiac event (MACE), in patients treated with BES, EES, and ZES. Study definitions used in this study are as follows: all-cause death (total death) including cardiac or noncardiac death. AMI was diagnosed as the presence of clinical symptoms, electrocardiographic changes, abnormal imaging findings of MI at angiography, and an increase in troponin-T/troponin-I and CK-MB to greater than the 99th percentile of the upper normal limit. Any coronary revascularization included TLR, target vessel revascularization (TVR), and nontarget vessel revascularization (NTVR) during the 3-year follow-up period. TLR was defined as revascularization of the target lesion due to restenosis or reocclusion within the stent or 5 mm in and adjacent to the distal or proximal segment. TVR was defined as revascularization of the target vessel or any segment of the coronary artery. NTVR was defined as revascularization of any segment of the nontarget coronary artery. 2 TLF was defined as composite of cardiac death, recurrent MI, and TLR. MACE was defined as the composite of total death, recurrent MI, and any coronary revascularization. In addition, Modified American College of Cardiology/American Heart Association criteria were used to classify coronary lesion morphology [5].

### 2.3. PCI Procedure and Medical Treatment

The loading doses of antiplatelet agents were as follows [6, 7]: aspirin was 200 mg, clopidogrel was 300 to 6+00 mg, ticagrelor was 180 mg, and prasugrel was 60 mg. The maintenance dose antiplatelet agents were as follows: aspirin was 100 mg, clopidogrel was 75 mg, ticagrelor was 90 mg twice a day, and prasugrel was 10 mg. If the patient <60 kg, the maintenance dose of prasugrel was reduced to 5 mg per day because of the potentially increased bleeding risk as described in a previous study.

Heparin administration before the procedure is as follows. The dose of unfractionated heparin was 100 IU/kg during the procedure and the dose was reduced to 70 IU/kg when it was combined with low molecular weight heparin Enoxaparin (Clexane®, Bristol-Myers Squibb and Sanofi-Aventis), 1 mg/Kg, and twice a day for 3–5 days.

Coronary angiography and intervention were approached with the femoral or radial artery. The administration of platelet glycoprotein IIb/IIIa receptor blockers depended on the operators' discretion. If the patient had typical angina symptoms or signs and over 70% diameter restenosis was observed in coronary angiography, the operators decided revascularization.

In-hospital stay and after discharge medications included aspirin, clopidogrel, ticagrelor, prasugrel, beta blockers (BB), calcium channel blocker (CCB), angiotensin-converting enzyme inhibitor (ACEI), angiotensin receptor blockers (ARB), and lipid-lowering agents.

### 2.4. Statistical Analysis

Continuous variables are expressed as means with standard deviations. Differences among the three groups, such as BES, EES, and ZES, were evaluated by analysis of variance in normally distributed data and Kruskal–Wallis H test in nonnormally distributed data. Post hoc analysis among the three groups was done using the Scheffe test or Dunnett-T3 test. Discrete variables are expressed as counts and percentages and the differences were analyzed with the *χ*^2^ test.

To adjust for any potential confounders, an inverse probability of treatment weighting (IPTW) analysis was performed [8, 9]. We utilized generalized boosted models to estimate the propensity score weight of each treatment using methods developed for the comparison of multiple treatments. The average treatment effect on the population weights was estimated using the multinomial propensity scores function in the Twang package in *R* Statistical Software (R Foundation for Statistical Computing, Vienna, Austria). We tested all available variables that could be of potential relevance: age, sex (male), left ventricular ejection fraction, cardiovascular risk factors (e.g., hypertension, diabetes, dyslipidemia, and stroke), comedication treatment (e.g., aspirin, other antiplatelets, RAS inhibitors, calcium channel blockers, beta blockers, and statins), angiographic and procedural characteristics (e.g., target vessel, a number of diseased vessels, and DES type). Clinical outcomes including total death, cardiac death, recurrent MI, TLR, ST, TLF, and MACE are estimated by Cox-proportional hazards models analysis. Binary logistic regression analysis is used to assess the hazard ratio (HR) of the BES group and ZES user group compared to the EES group in the IPTW population. A two-tailed *P* value of <0.05 was considered statistically significant. SPSS software, version 20 (SPSS Inc., Chicago, IL, USA), and R statistical software are used for statistical analysis.

## 3. Results

Baseline characteristics are listed in Table 1. The mean values of left ventricular ejection fraction were higher in the BES group than in the other two DES groups. The rates of previous diabetes mellitus (DM), stroke, PCI, and coronary artery bypass graft (CABG) were lower in the BES group than in the other two. The use of clopidogrel was lower in the EES group, while the use of ticagrelor was higher in that group compared to the other two groups. The use of aspirin, clopidogrel, prasugrel, and statin was lower in the EES group, while the use of ticagrelor was higher in that group compared to the other two groups. Aspirin and prasugrel were used more in the BES group, but ticagrelor was used less in the BES group compared to the other two DES groups. However, these intergroup differences in baseline characteristics were well balanced after IPTW adjustment, except the use of prasugrel, was used more in the BES group than the other groups.

Procedural characteristics are also listed in Table 1. The number of stents and the rates of left anterior descending (LAD) artery, right coronary artery (RCA), left main (LM), and multivessel disease as treated vessels were lower in the BES group, and the stent length was shorter in the BES group than the other group. The number of stents and the rate of LAD, LM, and MVD were higher in the BES group, and the stent length was longer in the EES group than in the other groups. However, these intergroup differences were well balanced after IPTW adjustment, but the rate of MVD and the number of stents were lower in BES than in the other groups, and stent length was longer in the EES group than the other groups.

Clinical outcomes up to three years are listed in Table 2. The rates of total death, cardiac death, TLF, and MACE were lower in the BES group than those in other DES groups. However, recurrent MI, ST, and any revascularization, such as TLR, TVL, or non-TVR were all similar in patients of all three DES groups. After IPTW adjustment, the rate of total death, cardiac death, TLF, and MACE were still lower in the BES group, and the rate of recurrent MI was also lower in the BES group than in the other DES group. However, the rate of any revascularization such as TLR, TVR, or non-TVR, and stent thrombosis were similar to the three groups.

**Figure 2:**
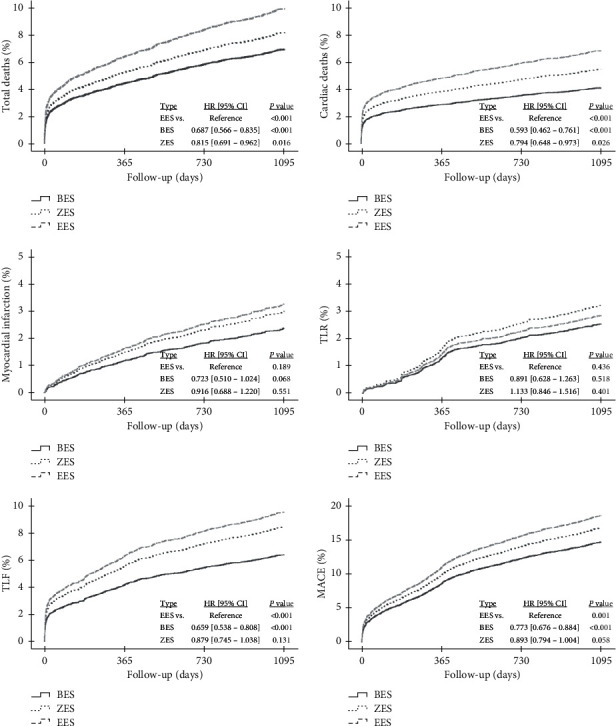
Inverse probability of treatment weighting score-adjusted survival curves from Cox-proportional hazards models for total death, cardiac death, recurrent MI, TLR, TLF, and MACE according to the type of DESs in the overall population. HR, hazard ratio; CI, confidence interval; BES, biolimus-eluting stent; EES, everolimus-eluting stent; ZES, zotarolimus-eluting stent; MI, myocardial infarction; TLR, target lesion revascularization; TLF, target lesion failure; MACE, major adverse cardiac event.

Clinical outcomes including total death, cardiac death, recurrent MI, TLF, and MACE are estimated by inverse probability of treatment weighting score-adjusted survival curves from Cox-proportional hazards models analysis according to the type of DESs such as BES, ZES, and EES in AMI patients (Figure 2). During the 3-year clinical follow-up, the BES group showed better outcomes of total death (HR, 0.687; 95% CI, 0.566–0.835; *P* < 0.001), cardiac death (HR, 0.593; 95% CI, 0.462–0.541; *P* < 0.001), TLF (HR, 0.659; 95% CI, 0.538–0.808; *P* < 0.001), and MACE (hazard ratio (HR), 0.773; 95% confidence interval (CI), 0.676–0.884; *P* < 0.001) as compared with EES groups.

## 4. Discussion

In this study, we compared the clinical outcomes up to three years among patients treated with BES, EES, or ZES. The interesting thing about this study compared to previous studies is that IPTW analysis was performed to adjust baseline potential confounders. After IPTW, at three years, total death, cardiac death, recurrent MI, TLF, and MACE were lowest in the BES group compared with the other DES groups in AMI patients. During the 3-year clinical follow-up, the BES group showed better outcomes of total death, cardiac death, TLF, and MACE than EES groups. The main reason for these results is that this study had a longer follow-up period and a larger number of enrolled patients than any previous study. Consequently, this research gives a clearer picture of the impact of BES on long-term clinical prognosis than was previously available.

Due to remarkable developments, next-generation DES continue to improve their clinical outcomes compared to previous generations [10]. However, in the case of AMI, there are still many challenges, and there are various opinions on the choice of DES [11].

The stent polymer of DES is a long-chain macromolecule component and plays a role in controlling drug release as a drug carrier vehicle. These polymers, however, can lead to undesirable biological responses. In the first-generation DES, durable polymers (DP) induce hypersensitivity and eosinophilic inflammatory reactions, resulting in delayed reendothelization of the vessels, increasing the incidence of ST [11]. Thus, inflammation caused by residual polymer eventually causes problems such as late stent malapposition, aneurysmal formation, and restenosis. Recently, there is great interest in DES or polymer-free DES coated with a biodegradable polymer (BP) for solving this problem.

BES is a polymer-free and carrier-free drug-coated stent that transfers umirolimus, which is also known as biolimus A9. Umirolimus is a highly lipophilic sirolimus analogue that transfers into the vessel wall over a period of one month [3]. In this study, we compared the three-year clinical outcomes of unrestricted use of BES and other DES in AMI patients in real-world clinical practice from 15 Korean PCI-capable institutions. Compared to all other groups, the BES group had a similar occurrence of revascularization and ST compared with the EES and ZES groups. These results were similar to previous studies using the KAMIR registry. Kim et al. reported that DP-DES, such as EES and ZES, and BP-DES, such as BES, showed comparable safety and efficacy during the two-year follow-up period [6]. Vlachojannis et al. have reported that BP-coated BES and DP-coated EES had similar safety and efficacy outcomes up to five years in the entire PCI population [12].

In this study, we compared three groups that showed significant differences in clinical characteristics at baseline. BES groups had higher left ventricular ejection fractions, lower rates of DM, previous MI, PCI, and CABG, and lower levels of troponin and serum creatinine than the other groups, and these differences in baseline characteristics have been associated with better outcomes of the BES group. There are several statistical methods for reducing the impact of confounding factors. Propensity-matched analysis is helpful in eliminating the bias in observational studies. In this study, we utilized generalized boosted models to estimate the propensity score weight of each treatment using IPTW analysis, and after IPTW, the three groups were balanced in baseline characteristics. We compared three stents to estimate the efficacy and safety of BES compared to the other stents. As a result, cardiac death, recurrent MI, TLF, and MACE were reduced in AMI patients treated with BES compared to the other DES stents. However, ST and any revascularization, such as TLR, TVL, or non-TVR, showed no differences among the patients of all three DES groups.

There are several limitations to this study. It is a nonrandomized design. We present the results of a multicentre observational registry comparing the outcome of BES versus other contemporary DESs, such as EES or ZES, in the setting of percutaneous coronary interventions for acute myocardial infarction. Therefore, each stent had a different duration of follow-up, and more missing data occurred than is typical in randomized studies. The choice of DES was dependent on the decision of each operator, which could affect the clinical outcome for that choice.

Second, the difference in vascular approaches has been reported to affect the complications and the access-site bleeding of patients in the ACS setting and impact the prevention of further cardiovascular events. However, this study produced no data relevant to the type of vascular approach used.

In conclusion, we compared the three DES groups such as BES, EES, or ZES and matched the difference of baseline characteristics using IPTW analysis. After IPTW, BES showed superior efficacy to EES in reducing total death, cardiac death, TLF, and MACE in AMI patients and similar efficacy to EES or ZES in revascularization and ST.

To get a final conclusion among the three DES groups, we assumed that a large, prospective, randomized controlled study is needed in the future.

## Figures and Tables

**Table 1:** Baseline clinical, angiographic, and procedural characteristics of acute myocardial infarction patients according to the use of DES type.

Variables	Entire population				IPTW weighted population			
	BES (*n* = 2,020)	ZES (*n* = 2,446)	EES (*n* = 5,293)	*P* value	BES (*n* = 6,036)	ZES (*n* = 5,795)	EES (*n* = 7,936)	*P* value
Men	1540 (76.2)	1850 (75.6)	3984 (75.2)	0.687	4588 (76.0)	4403 (75.9)	5994 (75.5)	0.754
Age (years)	63.3 ± 12.5	63.7 ± 12.3	64.1 ± 12.2	0.358	63.9 ± 12.2	64.2 ± 12.2	64.0 ± 12.2	0.245
LVEF (%)	53.1 ± 10.6	52.1 ± 10.6	51.4 ± 10.7	0.022	52.0 ± 10.5	52.2 ± 10.7	51.7 ± 10.6	0.065
BMI (Kg/m^2^)	24.0 ± 3.2	24.2 ± 3.1	24.0 ± 3.3	0.144	24.1 ± 3.1	24.1 ± 3.0	24.0 ± 3.2	0.081
STEMI	1043 (51.6)	1291 (52.7)	2772 (52.3)	0.743	3122 (51.7)	3071 (52.9)	4156 (52.3)	0.384
NSTEMI	977 (48.3)	1155 (47.2)	2521 (47.6)		2914 (48.2)	2724 (47)	3780 (47.6)	
Hypertension	971 (48.0)	1205 (49.2)	2675 (50.5)	0.148	2886 (47.8)	2878 (49.6)	3945 (49.7)	0.053
Diabetes mellitus	498 (24.6)	704 (28.7)	1500 (28.3)	0.003	1609 (26.6)	1632 (28.1)	2197 (27.6)	0.169
Dyslipidemia	224 (11.0)	299 (12.2)	593 (11.2)	0.364	705 (11.6)	640 (11.0)	874 (11.0)	0.405
Previous stroke	85 (4.2)	118 (4.8)	300 (5.6)	0.029	272 (4.5)	268 (4.6)	408 (5.1)	0.169
Previous HF	21 (1.0)	23 (0.9)	60 (1.1)	0.737	48 (0.7)	63 (1.0)	86 (1.0)	0.168
Previous PCI	104 (5.1)	217 (8.8)	471 (8.8)	<0.001	449 (7.4)	467 (8.0)	667 (8.4)	0.112
Previous CABG	6 (0.2)	8 (0.3)	38 (0.7)	0.024	41 (0.6)	17 (0.2)	48 (0.6)	0.710
Smoking history	1216 (60.1)	1467 (59.9)	3110 (58.7)	0.413	3613 (59.8)	3485 (60.1)	4719 (59.4)	0.721
Current smoker	844 (41.7)	1009 (41.2)	2093 (39.5)	0.139	2478 (41.0)	2340 (40.3)	3199 (40.3)	0.636
Exsmoker	372 (18.4)	458 (18.7)	1017 (19.2)	0.707	1135 (18.8)	1144 (19.7)	1521 (19.1)	0.424

Laboratory findings								
HbA1c (%)	6.4 ± 1.4	6.5 ± 1.4	6.5 ± 1.5	<0.001	6.3 ± 1.3	6.4 ± 1.4	6.4 ± 1.4	0.088
TC (mg/dL)	181 ± 44	182 ± 44	179 ± 45	0.389	179 ± 42	180 ± 43	180 ± 44	0.457
LDL (mg/dL)	115 ± 37	114 ± 39	114 ± 39	0.854	114 ± 37	114 ± 39	114 ± 38	0.514

Discharge medication								
Aspirin	1995 (98.7)	2409 (98.4)	5175 (97.7)	0.007	5944 (98.4)	5699 (98.3)	7776 (97.9)	0.070
Clopidogrel	1351 (66.8)	1723 (70.4)	3533 (66.7)	0.004	4028 (66.7)	3967 (68.4)	5350 (67.4)	0.134
Cilostazol	198 (9.8)	255 (10.4)	503 (9.5)	0.447	589 (9.7)	620 (10.6)	778 (9.8)	0.150
Prasugrel	280 (13.8)	244 (9.9)	520 (9.8)	<0.001	686 (11.3)	581 (10.0)	815 (10.2)	0.037
Ticagrelor	359 (17.7)	440 (17.9)	1114 (21.0)	<0.001	1189 (19.7)	1139 (19.6)	1596 (20.1)	0.755
CCB	100 (4.9)	154 (6.2)	308 (5.8)	0.152	307 (5.0)	341 (5.8)	463 (5.8)	0.096
Beta blockers	1713 (84.8)	2092 (85.5)	4473 (84.5)	0.509	5127 (84.9)	4941 (85.2)	6724 (84.7)	0.687
ACEI or ARB	1636 (80.9)	1962 (80.2)	4241 (80.1)	0.698	4786 (79.3)	4686 (80.8)	6388 (80.4)	0.080
Statins	1889 (93.5)	2297 (93.9)	4891 (92.4)	0.033	5616 (93.0)	5453 (94.0)	7364 (92.7)	0.420

*Angiographic and procedural characteristics*								
Treated artery								
LAD	1161 (57.4)	1437 (58.7)	3217 (60.7)	0.023	3594 (59.5)	3450 (59.5)	4761 (59.9)	0.816
LCX	559 (27.6)	651 (26.6)	1459 (27.5)	0.639	1611 (26.6)	1547 (26.6)	2176 (27.4)	0.534
RCA	762 (37.7)	1043 (42.6)	2166 (40.9)	0.003	2437 (40.3)	2463 (42.5)	3246 (40.9)	0.050
LM	72 (3.5)	128 (5.2)	287 (5.4)	0.004	256 (4.2)	271 (4.6)	396 (4.9)	0.116
MVD	877 (43.4)	1304 (53.3)	2860 (54.0)	<0.001	3003 (49.7)	3018 (52.0)	4131 (52.0)	0.011
Stent number	1.58 ± 0.73	1.73 ± 0.77	1.75 ± 0.78	<0.001	1.68 ± 0.77	1.71 ± 0.77	1.72 ± 0.77	0.047
Stent *D* (mm)	3.12 ± 0.40	3.13 ± 0.42	3.11 ± 0.42	0.104	3.12 ± 0.42	3.13 ± 0.42	3.12 ± 0.41	0.436
Stent *L* (mm)	21.8 ± 9.6	25.1 ± 12.4	27.3 ± 13.2	0.007	24.9 ± 12.6	24.5 ± 12.5	25.9 ± 12.7	0.001

IPTW, inverse probability of treatment weighting; BES, biolimus-eluting stent; EES, everolimus-eluting stent; ZES, zotarolimus-eluting stent; LVEF, left ventricular ejection fraction; BMI, body mass index; STEMI, ST-elevation myocardial infarction; NSTEMI, non-ST-elevation myocardial infarction; HF, heart failure; PCI, percutaneous coronary intervention; CABG, coronary artery bypass graft; TC, total cholesterol; LDL, low-density lipoprotein; CCB, calcium channel blocker; ACEI, angiotensin-converting enzyme inhibitor; ARB, angiotensin receptor blocker; LAD, left anterior descending coronary artery; LCX, left circumflex coronary artery; RCA, right coronary artery; LM, left main artery; *D*, diameter; *L*, length.

**Table 2:** Clinical outcomes of acute myocardial infarction patients with preserved left ventricular systolic function according to the use of DES type during a 3-year follow-up.

Variables	Entire population				IPTW weighted population			
	BES (*n* = 2020)	ZES (*n* = 2446)	EES (*n* = 5293)	*P* value	BES (*n* = 6036)	ZES (*n* = 5795)	EES (*n* = 7936)	*P* value
MACE	303 (15.0)	415 (16.9)	988 (18.6)	0.001	959 (15.8)	1017 (17.5)	1446 (18.2)	0.001
TLF	127 (6.2)	204 (8.3)	500 (9.4)	<0.001	393 (6.5)	472 (8.1)	730 (9.1)	<0.001
Total death	141 (6.9)	201 (8.2)	526 (9.9)	<0.001	436 (7.2)	499 (8.6)	761 (9.5)	<0.001
Cardiac death	82 (4.0)	133 (5.4)	361 (6.8)	<0.001	253 (4.1)	312 (5.3)	525 (6.6)	<0.001
Recurrent MI	44 (2.1)	69 (2.8)	168 (3.1)	0.073	102 (1.6)	152 (2.6)	254 (3.2)	<0.001
STEMI	9 (0.4)	12 (0.4)	45 (0.8)	0.073	21 (0.3)	17 (0.2)	64 (0.8)	<0.001
NSTEMI	35 (1.7)	58 (2.3)	123 (2.3)	0.255	81 (1.3)	139 (2.3)	191 (2.4)	<0.001
Revascularization	162 (8.0)	213 (8.7)	453 (8.5)	0.685	527 (8.7)	510 (8.8)	670 (8.4)	0.724
TLR	46 (2.2)	71 (2.9)	138 (2.6)	0.427	142 (2.3)	142 (2.4)	204 (2.5)	0.710
TVR	93 (4.6)	108 (4.4)	243 (4.5)	0.934	294 (4.8)	244 (4.2)	363 (4.5)	0.226
Non-TVR	79 (3.9)	110 (4.4)	219 (4.1)	0.606	263 (4.3)	272 (4.6)	319 (4.0)	0.156
Stent thrombosis	10 (0.4)	10 (0.4)	27 (0.5)	0.832	23 (0.3)	32 (0.5)	40 (0.5)	0.375

IPTW, inverse probability of treatment weighting; BES, biolimus-eluting stent; EES, everolimus-eluting stent; ZES, zotarolimus-eluting stent; MACE, major adverse cardiac event, the composite of total death, recurrent MI and revascularization; TLF, target lesion failure, composite of cardiac death, recurrent MI, and TLR; MI, myocardial infarction; STEMI, ST-elevation myocardial infarction; NSTEMI, non-ST-elevation myocardial infarction; TLR, target lesion revascularization; TVR, target vessel revascularization.

## Data Availability

All relevant data can be assessed from the KAMIR website via the following URL: http://www.kamir.or.kr.

## References

[1] Tada T., Byrne R. A., Simunovic I. (2013). Risk of stent thrombosis among bare-metal stents, first-generation drug-eluting stents, and second-generation drug-eluting stents. *JACC: Cardiovascular Interventions*.

[2] Kim Y. H., Her A.-Y., Jeong M. H. (2019). A comparison of the impact of current smoking on 2-year major clinical outcomes of first- and second-generation drug-eluting stents in acute myocardial infarction. *Medicine*.

[3] Kogame N., Chichareon P., De Wilder K. (2020). Clinical relevance of ticagrelor monotherapy following 1‐month dual antiplatelet therapy after bifurcation percutaneous coronary intervention: insight from GLOBAL LEADERS trial. *Catheterization and Cardiovascular Interventions*.

[4] Jensen C. J., Naber C. K., Urban P. (2018). Two-year outcomes of high bleeding risk patients with acute coronary syndrome after Biolimus A9 polymer-free drug-coated stents: a LEADERS FREE substudy. *EuroIntervention*.

[5] O’Gara P. T., Kushner F. G., Ascheim D. D. (2013). ACCF/AHA guideline for the management of ST-elevation myocardial infarction: a report of the American College of Cardiology foundation/American heart association task force on practice guidelines. *Circulation*.

[6] Kim Y. H., Her A.-Y., Jeong M. H. (2018). Impact of current smoking on 2-year clinical outcomes between durable-polymer-coated stents and biodegradable-polymer-coated stents in acute myocardial infarction after successful percutaneous coronary intervention: data from the KAMIR. *PloS One*.

[7] Park J. Y., Choi B. G., Rha S.-W., Kang T. S. (2018). Five-year outcomes in patients with anemia on admission undergoing a coronary intervention for acute myocardial infarction in Koreans. *Coronary Artery Disease*.

[8] Sim D. S., Jeong M. H., Kim H. S. (2020). Dual antiplatelet therapy beyond 12 months versus for 12 months after drug-eluting stents for acute myocardial infarction. *Journal of Cardiology*.

[9] Kim K.-H., Choi B. G., Choi B. G., Rha S.-W., Choi C. U., Jeong M.-H. (2021). Impact of renin angiotensin system inhibitor on 3-year clinical outcomes in acute myocardial infarction patients with preserved left ventricular systolic function: a prospective cohort study from Korea Acute Myocardial Infarction Registry (KAMIR). *BMC Cardiovascular Disorders*.

[10] Borhani S., Hassanajili S., Ahmadi Tafti S. H., Rabbani S. (2018). Cardiovascular stents: overview, evolution, and next generation. *Progress in Biomaterials*.

[11] Kim Y. H., Her A. Y., Jeong M. H. (2020). Impact of stent generation on 2-year clinical outcomes in ST-segment elevation myocardial infarction patients with multivessel disease who underwent culprit-only or multivessel percutaneous coronary intervention. *Journal of the Society for Cardiac Angiography & Interventions*.

[12] Vlachojannis G. J., Smits P. C., Hofma S. H. (2017). Biodegradable polymer biolimus-eluting stents versus durable polymer everolimus-eluting stents in patients with coronary artery disease. *JACC: Cardiovascular Interventions*.

